# SRP and NAC: Guiding a Nascent Protein’s First Steps

**DOI:** 10.1371/journal.pbio.1001103

**Published:** 2011-07-12

**Authors:** Caitlin Sedwick

**Affiliations:** Freelance Science Writer, San Diego, California, United States of America

**Figure:**
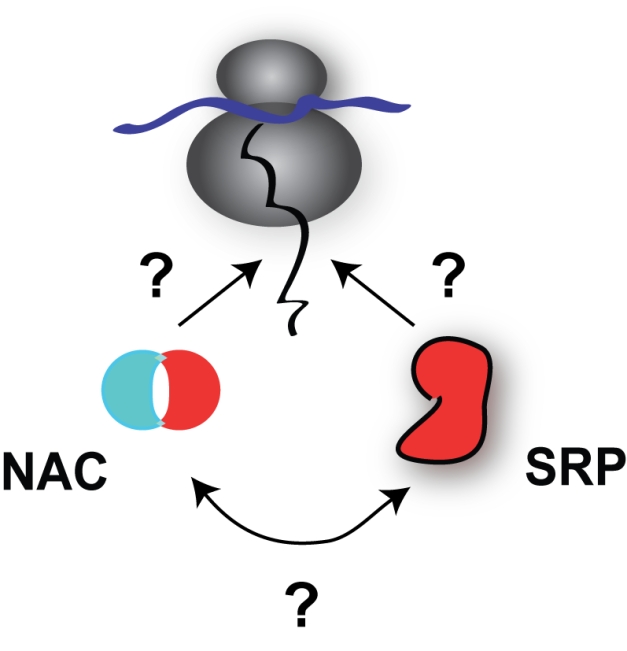
A novel method to understand how ribosome-associated factors recognize the “right” nascent protein, directing them to their correct cellular location and fate, sheds light on the specificity and interplay of the membrane-targeting SRP complex and the cytosolic chaperone NAC.

A newly synthesized protein is not ready to start working in a cell immediately after it is made by the ribosome. Instead, these nascent proteins must first fold into their proper 3-D shape, and possibly also receive modifications such as the addition of sugars or lipids. The site where these maturation steps take place depends upon where the final protein will be used in the cell: cytosolic proteins fold in the cytosol, whereas mitochondrial proteins fold in mitochondria, and secreted proteins and transmembrane proteins must first be shuttled to and threaded through the endoplasmic reticulum (ER) membrane before they can fold. How are nascent proteins sorted and shepherded to the proper site for folding? That’s the question that Marta del Alamo, Judith Frydman, and colleagues set out to explore in their paper published in *PLoS Biology* this month.

Not much is known about the recognition and trafficking of nascent proteins. One thing that is known is that, as proteins emerge from the ribosome, many of those that are destined for folding in the ER are shuttled there by a protein–RNA complex called the signal recognition particle (SRP). SRP is thought to recognize and bind to those proteins that possess either a special amino acid sequence (called the signal sequence), or the stretches of hydrophobic amino acids that typify transmembrane domains. SRP’s binding properties had been inferred from experiments that detailed its interactions with relatively few proteins; however, they had never been surveyed systematically. Alamo and colleagues therefore decided to investigate which of the many proteins made in a cell are bound by SRP, and whether other factors also modulate the earliest protein trafficking steps.

Because new proteins emerge from ribosomes one amino acid at a time, the mRNAs that encode those proteins remain attached to their ribosomes as they are being translated. The authors reasoned that by immunoprecipitating the ribosomes they would simultaneously fish out all the mRNAs being translated in the cell, which could then be identified using DNA microarrays. And, because SRP directly binds to translating ribosomes, immunoprecipitating SRP would allow the identification of the subset of mRNAs that encode SRP’s target proteins.

By applying this approach to yeast cells and analyzing the corresponding mRNAs, the group was able to determine what sets SRP’s targets apart from all the other mRNAs being translated in the cells. The experiment confirmed earlier observations: SRP is mostly associated with proteins that have a transmembrane domain or a signal sequence, and that are destined for membranous structures or secretion, respectively. But, it also turned up some SRP substrates that have neither transmembrane nor signal sequences. Additionally, it showed that some secretory proteins do not associate with SRP, indicating that there is more to learn about what dictates whether SRP binds to a particular protein.

One thing that may help explain SRP’s target specificity would be the presence of other proteins that modulate SRP’s interactions with its substrates. A protein called nascent chain associated complex (NAC)—less well characterized than SRP—might perform such a role.

When the researchers examined NAC’s substrates by using a similar approach as for SRP, they found that NAC was associated with practically every translating ribosome —and therefore every nascent protein—in the cell. But, there are three different forms of NAC in yeast cells and each form has different substrate specificities: one form is found on ribosomes translating mitochondrial proteins or ribosomal proteins, whereas the other two associate with ribosomes translating secretory pathway proteins. These forms of NAC were found to interact with some of the same nascent secretory pathway proteins at the same time as SRP. What’s more, experiments in NAC-deficient cells showed that some proteins require NAC in order to interact with SRP, whereas others are prevented from interacting with SRP when NAC is present. NAC therefore appears to be important for modulating the interactions of nascent proteins with SRP. Nonetheless, the group showed, yeast cells lacking NAC do not suffer from large impairments in targeting proteins to their appropriate compartments for folding. That’s because other chaperone proteins can substitute when NAC is absent.

Collectively, these data provide new insights into the functions of both SRP and NAC, and into how the early steps of protein production are managed. And, Alamo and colleagues are already employing the experimental approaches used in this paper to uncover yet more new findings about this critical process.

**del Alamo M, Hogan DJ, Pechmann S, Albanese V, Brown PO, et al. (2011) Defining The Specificity of Cotranslationally Acting Chaperones by Systematic Analysis of mRNAs Associated with Ribosome-Nascent Chain Complexes. doi:10.1371/journal.pbio.1001100**

